# Determination of viable myocardium through delayed enhancement cardiac magnetic resonance imaging combined with ^18^F-FDG PET myocardial perfusion/metabolic imaging before CABG

**DOI:** 10.1007/s10554-024-03057-3

**Published:** 2024-01-24

**Authors:** Dongsheng Xu, Jiwang Zhang, Bing Liu, Donghai Fu, Jianming Li, Lijuan Fan

**Affiliations:** 1 Department of Radiology, TEDA International Cardiovascular Hospital, Tianjin, 300457 China; 2 Department of Nuclear Medicine, TEDA International Cardiovascular Hospital, Tianjin, 300457 China; 3 Tianjin Key Laboratory of Molecular Regulation of Cardiovascular Diseases and Translational Medicine, Tianjin, 300457 China

**Keywords:** DE-CMR, ^18^F-FDG PET, Myocardial perfusion imaging, Metabolic imaging, Coronary artery bypass grafting

## Abstract

Purpose: Study aims to investigate the consistency of delayed enhancement cardiac magnetic resonance imaging (DE-CMR) and ^18^F-FDG PET myocardial imaging in evaluating myocardial viability before CABG. Methods: The study analyzed data from 100 patients who were examined with DE-CMR, PET imaging, and echocardiography before and after CABG. All subjects were followed up for 6–12 month post- CABG. Results: DE-CMR and PET imaging have high consistency (90.1%; Kappa value = 0.71, *p* < 0.01) in determining myocardial viability. The degree of delayed enhancement was negatively correlated with the improvement in myocardial contractile function in this segment after revascularization (*P* < 0.001). The ratio of scarred myocardial segments and total DE score was significantly lower in the improvement group than non-improvement group. Multivariate regression identified that hibernating myocardium (OR = 1.229, 95%CI: 1.053–1.433, *p* = 0.009) was influencing factor of LVEF improvement after CABG. Conclusion: Both imaging techniques are consistent in evaluating myocardial viability. Detecting the number of hibernating myocardium by PET is also important to predict the left heart function improvement after CABG.

## Introduction

Coronary artery bypass grafting (CABG) effectively treats severe coronary atherosclerotic heart disease (left main trunk and/or triple vessel diseases) and improves symptoms in patients with cardiac insufficiency. Patients receiving CABG treatment have lower mortality rate, rate of major adverse cardiac events (MACE), and rate of target vessel revascularization than those receiving PCI treatment [1, 2]. The CABG treatment group also had lower heart failure hospitalization rate over three years compared to the PCI group [3]. The success of CABG is associated with the proportion of surviving myocardium [4], as improved postoperative cardiac function occurs only when blood supply to viable myocardium returns to normal after revascularization. This then results in improved left ventricle ejection fraction (LVEF), clinical symptoms, long-term survival, and the effect of revascularization can be fully utilized. However, for the patients without viable myocardium, CABG provides little improvement [5, 6], resulting in high risk of death [7]. In addition, surgeries could lead to trauma and complications, and possibly worsen the patient’s condition.

Viable myocardium refers to dysfunctional myocardial tissue that is still capable of surviving despite systolic dysfunction and either a reduction (hibernation) or non-reduction (stunning) in myocardial perfusion. These cells still possess intact membrane integrity, cellular metabolism, and the potential contraction reserve [8]. Currently, several imaging methods are available for detecting viable myocardium mainly include dobutamine stress echocardiography, single-photon emission computed tomographic myocardial perfusion imaging (SPECT), myocardial metabolism imaging (including oxygen metabolism, fatty acid metabolism, and glucose metabolism), and delayed enhancement cardiac magnetic resonance imaging (DE-CMR). DE-CMR has been considered as the “gold standard”, it can reliably detect both viable and scarred myocardium. The ^18^F-FDG PET myocardial perfusion-metabolic imaging is an internationally recognized “gold standard” technique to identify viable myocardium. Previous studies have shown significantly higher sensitivity of hibernating myocardium and non-transmural myocardial scar in predicting overall cardiac function improvement than collateral blood flow and other indicators [9]. Moreover, studies have demonstrated high correlation and consistency between these two methods in evaluating myocardial viability [10–13].

Hence, wise combination of both ^18^F-FDG PET and DE-CMR techniques prior to CABG to precisely assess the condition of myocardium, as well as structure and function of heart. The accurate evaluation of these parameters holds significant importance in preoperative screening and predicting the prognosis of patients. Therefore, this study aims to comprehensively compare the efficiency of DE-CMR and ^18^F-FDG PET myocardial perfusion-metabolic imaging in predicting the improvement of left heart function post CABG surgery. By identifying the pros and cons of each method, we can determine the optimal approach for preoperative evaluation and patient’s management during surgery.

## Materials and methods

### Subjects and study design

This study retrospectively analyzed data from 100 patients with left main trunk disease/multi-vessel diseases accompanied with cardiac insufficiency. The patients were selected by coronary angiography at the Heart Center of Teda International Cardiovascular Hospital from June 2018 to June 2021.All the participants underwent DE-CMR, ^13^N-NH_3_/^18^F-FDG PET myocardial perfusion-metabolic imaging, and echocardiography within two weeks prior to CABG procedure. Follow up examinations were conducted from six months to one year after the procedure, and echocardiography was done to evaluate LVEF. CABG recommendations from the 2011 ACCF/AHA guidelines were as follows: (1) Left main disease greater than 50%; (2) Three-vessel coronary artery disease of greater than 70% with or without proximal LAD involvement; (3) Two-vessel disease: LAD plus one other major artery; (4) One or more significant stenosis greater than 70% in a patient with significant anginal symptoms despite maximal medical therapy; 3) One vessel disease greater than 70% in a survivor of sudden cardiac death with ischemia-related ventricular tachycardia. The study inclusion criteria were as follows: (1) Patients having CHD, left main trunk and/or multi-vessel diseases proved by coronary DSA or CT coronary imaging; (2) having a history of myocardial infarction; and (3) preoperative LVEF < 50% [14], and New York Heart Association (NYHA) ≥ Grade II. The exclusion criteria were as follows: (1) Patients with dilated or hypertrophic cardiomyopathy, or other non-ischemic cardiomyopathy; (2) have rheumatic heart disease; (3) Contraindications to MRI examination (such as metal implants or claustrophobia). The study was approved by the Ethics Committee of the Teda International Cardiovascular Hospital, land all patients signed informed consent.

### Imaging method

The images in this study were captured by GE1.5T MRI Scanner, as well as specialized GE cardiac coil was used for the DE-CMR. To conduct first-pass myocardial perfusion and delayed enhancement imaging, we utilized ECG gating technology and breath-holding technology. For ^13^N-NH_3_/^18^F-FDG PET myocardial perfusion-metabolic imaging, our team employed the GE Discovery Elite PET/CT and GE Qilin Medical cyclotron. The positron drugs were self-designed by the Department of Nuclear Medicine. The ^13^N-NH_3_. H_2_O PET myocardial perfusion imaging was completed on the first day, and the ^18^F-FDG PET myocardial metabolic imaging was performed next day. We performed echocardiography using the GE VividE9 color Doppler ultrasound diagnostic instrument.

### Data collection and analysis

#### DE-CMR

The heart function-specific analysis software (ReportCARD4.0, GE) was used toperform image analysis. The short axis plane was aligned with the long axis image of the two-chamber view and adjusted based on the location of the papillary muscle. Specifically, the middle segment aligned with the level of the papillary muscle, while the basal and apical segments respectively corresponds to the levels above and below the papillary muscle. All myocardial segments were visually evaluated and scored using a 4-level method [15] ,0 point indicating normal movement; 1 point indicating reduced movement; 2 points indicating no movement; and 3 points indicating contradictory movement. The degree of myocardial enhancement delay (DE) was semi-quantified by 5 level system corresponding 17-segment model for left ventricular myocardial segmentation recommended by the American Heart Association (AHA) [16]. DE scored as follows: 0 point indicating no enhancement; 1 point indicated 1-25% enhancement; 2 points indicated 26-50% enhancement; 3 points indicated 51-75% enhancement; and 4 points indicated ≥ 76% enhancement. To conduct a comparative analysis, relevant literature was used to define following indicators [17]: (1) involved segment: denoted the number of segments exhibiting delayed enhancement (DE); (2) viable myocardial segments: segments with scores falling within the range of 0, 1, or 2 points; (3) segments of scarred myocardium: segments with the scores of 3 or 4 points; and (4) total score of DE: the total DE score of all segments.

### ^18^F-FDG PET myocardial perfusion-metabolic imaging

For deep image analysis, images were transferred to the specialized workstation (Xeleris 2.0), where Myovation and ECToolbox softwares were used for image reconstruction and quantitative post-processing. The OSEM method was implemented for image reconstruction, which could show the horizontal long-axis, vertical long-axis, and short-axis images of the heart. The polar bull’s-eye plot was reconstructed using the short-axis image. The evaluation of images followed the 17-segment method as per AHA recommendations [18]. Image score as follows, 0:normal uptake; 1:slightly reduced uptake; 2: moderately reduced uptake; 3: severely reduced uptake; and 4: no uptake. Based on the score obtained from myocardial perfusion-metabolic imaging, the viability of myocardium was evaluated [18]. Viable myocardium comprised segments with the myocardial perfusion scores of 1, 2, or 3 points and segments with mismatch perfusion-metabolism when myocardial perfusion scores were 3 or 4 points (hibernating myocardium).Scarred myocardium denotes the segments with the myocardial perfusion scores of 3 or 4 points with matching perfusion-metabolism.

All CMR and PET data were collected, stored, and analyzed independently by two experienced radiologists and nuclear medicine physicians who were unaware of the clinical and imaging results.

### Echocardiography

The left ventricular end-diastolic dimension (LVEDD), left ventricular end-systolic volume (LVESV), and left ventricular end-diastolic volume (LVEDV) were measured on the parasternal long-axis view. The LVEF was calculated by the Simpsons method on the apical four-chamber view. All myocardial segments were visually evaluated and scored using a 4-level method [15]. Based on the preoperative and postoperative LVEF difference (△LVEF), the patients were categorized into the LVEF improvement group (△LVEF ≥ 5% group) and LVEF non-improvement group (△LVEF < 5% group) [19, 20].

### Baseline data

The baseline data of the subjects were obtained from the Case System and the PACS System of Heart Center of Teda International Cardiovascular Hospital.

### Statistical analysis

The study used SPSS21.0 software and statistical analysis (SPSS, USA) was done using mean, ± standard deviation and median (interquartile range). Independent t-test or U test was used for the comparisons between two groups. Kappa test evaluated the consistency between DE-CMR and PET myocardial imaging in evaluating myocardial viability. The rates were compared by the χ^2^ test. All parameters considered relevant for improvement of LVEF ≥ 5% were analyzed by univariate analysis, and then only significant parameters were into multivariate analysis. Binary logistic regression analysis was done to assess the association between postoperative LVEF improvement and indicators with statistical significance, and the odds ratio and corresponding 95% confidential interval was calculated. The receiver operator characteristic curve (ROC) was plotted, the area under curve (AUC) was calculated, and the cut-off value of number of hibernating myocardium in improving the LVEF after CABG was estimated. Statistically significant was considered as *p* < 0.05.

## Results

### General characteristics of patients

The details of clinical and imaging data of participants are provided in Table 1. All the analyses were performed on data from 90 patients excluding 10 due to death or poor image quality (2 died of postoperative cerebrovascular accident, 1 died of multi-organ failure, 2 died of recurrent acute myocardial infarction, and 5 were excluded for poor quality of images). Out of the 90 patients, 70 were males and 20 were females, with a mean age of 61.44 ± 9.13 years. Table 1 shows the LVEDV, LVESV, and LVEF measured by DE-CMR and PET myocardial perfusion-metabolic imaging before CABG. The results showed that the evaluation of LVEDV and LVESV by these two methods was significantly different (*p* = 0.000), but the evaluation of LVEF was not significantly different between the two methods (*p* = 0.254). The accumulation score was not significantly different between DE-CMR and PET myocardial perfusion-metabolic imaging(*p* = 0.133,Table 1).

**Table 1:** Baseline characteristics and imaging findings of patients

Indicators	Value
Age (years)	61.44 ± 9.13
Sex (M/F)	70/20
History of myocardial infarction (n, %)	90(100)
Diabetes (n, %)	32(35.6)
Hypertension (n, %)	55(61.1)
Hyperlipidemia (n, %)	29(32.2)
Smoking history (n, %)	43(47.8)
History of previous PCI (n, %)	65(72.2)
Coronary stenosis > 50%	
Left main trunk (n, %)	18(20)
Double vessel disease (n, %)	33(36.7)
Triple vessel disease (n, %)	57(63.3)
AHA heart function grading	2.20 ± 0.64
DE-CMR	
LVEF (%)	30(11)
LVEDV (mL)	228(81.25)
LVESV (mL)	162(68.75)
Total score of delayed enhancement (points)	19.89 ± 11.14
PET myocardial perfusion-metabolic imaging	
LVEF (%)31(13)	
LVEDV (mL)	169.50 (78)
LVESV (mL)	113.5(75)
Total score of abnormal uptake (points)	18.94 ± 9.50

Among 1530 myocardial segments examined by DE-CMR, 835 segments (54.6%) showed different degrees of delayed enhancement. According to the delayed enhancement imaging outcomes of DE-CMR revealed viable and scarred myocardium 75.6% (1157/1530) and 24.4% (373/1530), respectively. PET myocardial perfusion-metabolic imaging identified 81.7% (1250/1530) viable and 18.3% (280/1530) scarred myocardium. Using the PET myocardial perfusion-metabolic imaging results as reference, the sensitivity, specificity, positive predictive value, and negative predictive value of DE-CMR in identifying viable myocardium was 97.5%, 67.3%, 90.2%, and 89.6%, respectively (Tables 2 and 3).

**Table 2:** Scoring details of 1530 segments evaluated by DE-CMR imaging and PET myocardial imaging

PET imaging score (points)	DE-CMR score			Total
	0	1–2	3–4	
0	559	85	7	651
1–2	87	283	59	429
Perfusion/metabolism mismatch	45	69	56	170
3–4	4	25	251	280
Total	695	462	373	1530

**Table 3:** Correlation between the viable myocardium evaluated by DE-CMR imaging and PET myocardial imaging

PET perfusion-metabolic imaging	DE-CMR		Total
	Segment of scarred myocardium	Segment of viable myocardium	
Segment of scarred myocardium	251	29	280
Segment of viable myocardium	122	1128	1250

**Fig. 1:**
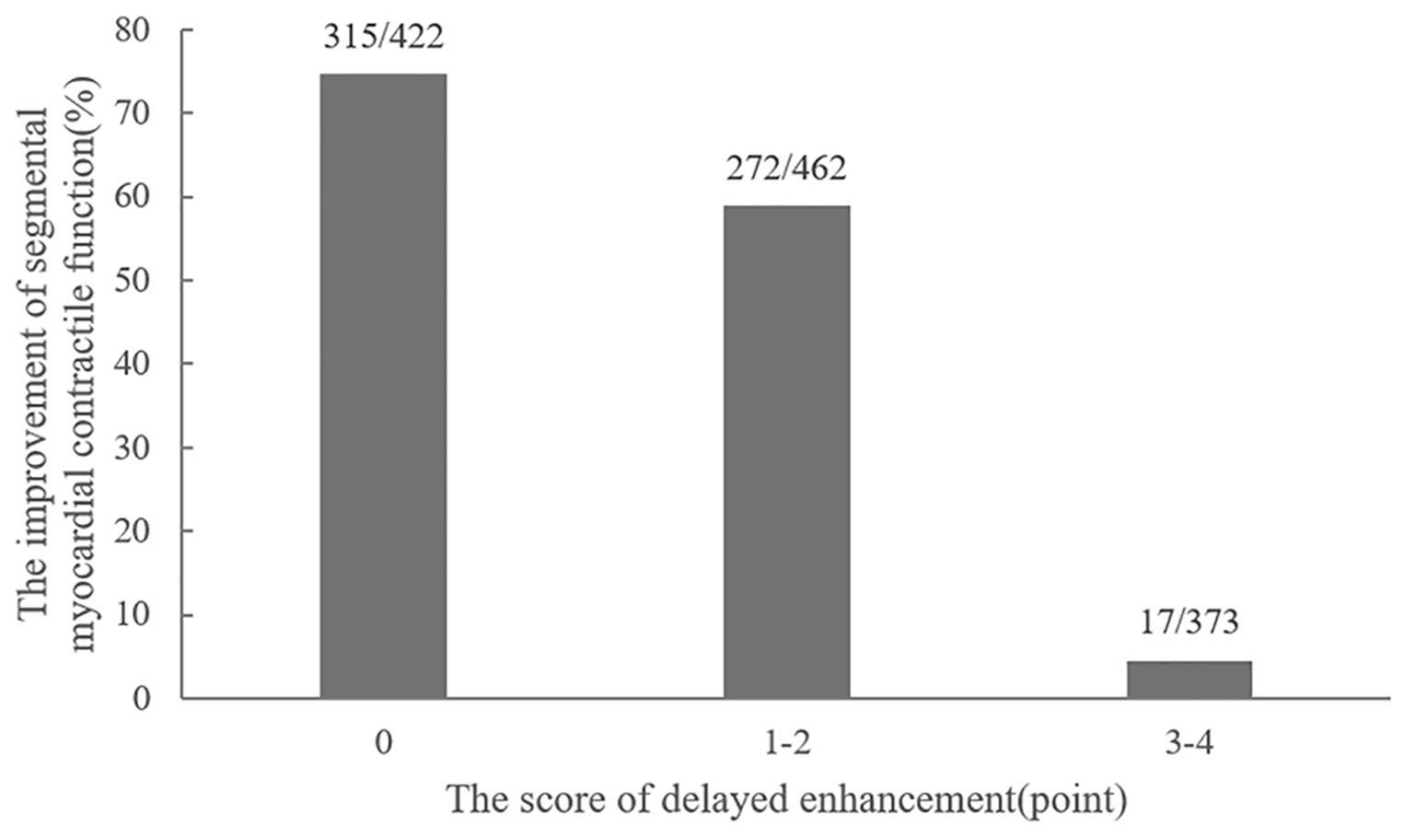
The relationship between the degree of delayed enhancement of segmental myocardium and myocardial contractile function

Of the 1530 myocardial segments evaluated by echocardiography before CABG, 1257 segments (82.2%) were identified as having abnormal motion, and 835 segments (54.6%) showed some degree of delayed enhancement. At 6 months to 1 year after CABG, 604 (315 + 272 + 17)/1257 (422 + 462 + 373) segments (48.1%) showed some degree of motion improvement. The degree of delayed enhancement was negatively correlated with the improvement in myocardial contractile function in this segment after revascularization (*P* < 0.001)(Fig. 1).

### Comparison of the findings of DE-CMR and PET myocardial perfusion-metabolic imaging

**Fig. 2:**
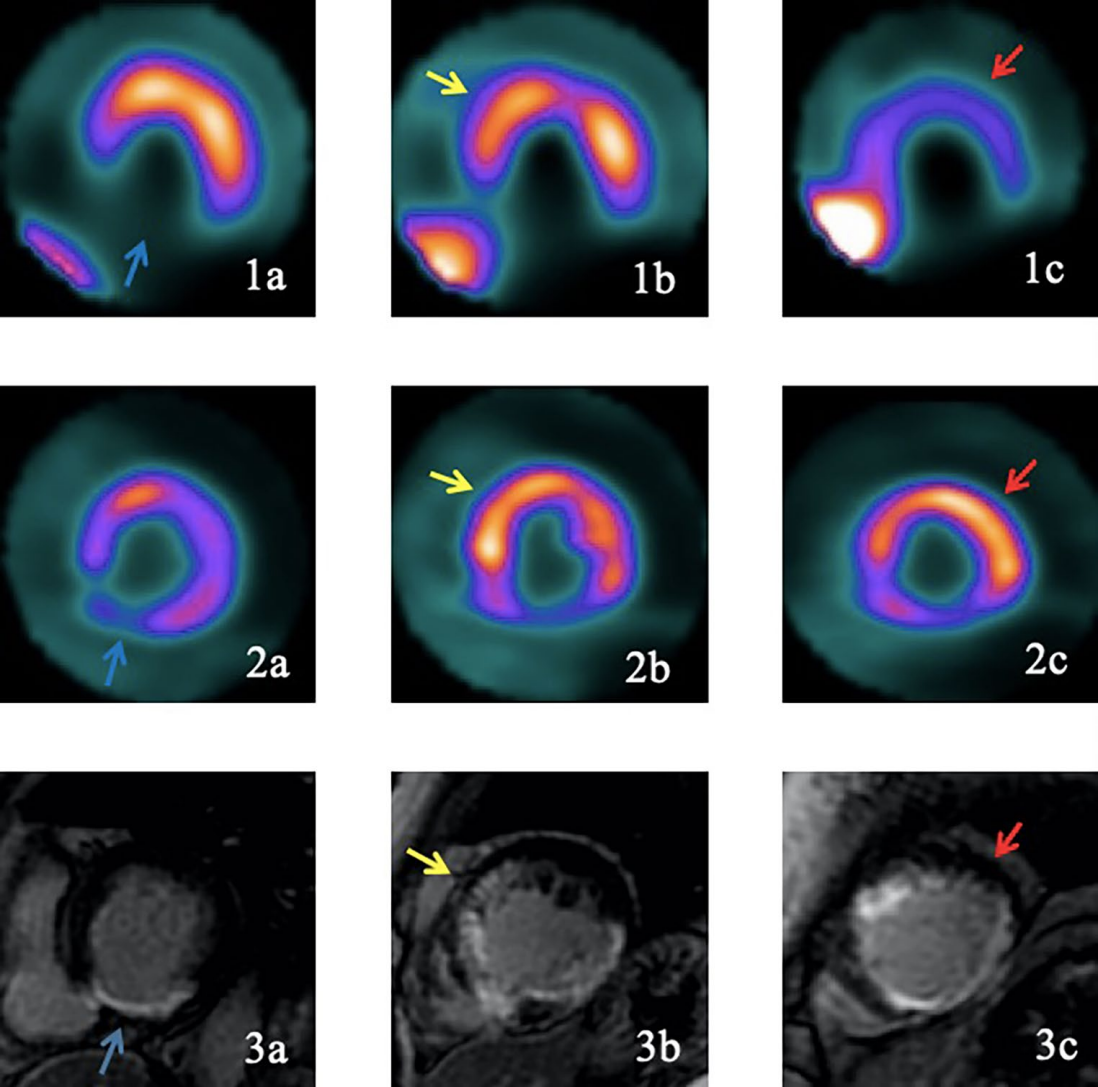
A 67-year-old male patient with coronary heart disease (double vessel disease), and the preoperative LVEF was 38%. The Fig. 2a and b, and 2c shows the ^13^N-NH_3_PET myocardial perfusion images; Fig. 3a and b, and 3c shows the ^18^F-FDG PET myocardial metabolic images; Fig. 1a and b, and 1c shows the DE-CMR images. All images showed short-axis view; a, left basal ventricle; b, left middle ventricle; and c, apex of the left ventricle. The PET myocardial perfusion and metabolism of lower wall of the basal left ventricle and part of the lower septal wall generally showed equally reduced uptake or no uptake (Figs. 2a and 3a). DE-CMR indicated transmural enhancement (Fig. 1a) (blue arrow). The perfusion and metabolism of the anterior septal wall of left ventricle were normal, indicating viable myocardium (Figs. 2b and 3b); while DE-CMR indicated transmural enhancement (Fig. 1b), and the findings of these two imaging methods were inconsistent (yellow arrows). The perfusion of anterior wall of the left ventricular apex was substantially reduced, while the metabolism was normal, and the perfusion/metabolism mismatch was present, indicating the presence of hibernating myocardium (Figs. 2c and 3c); DE-CMR indicated subendocardial enhancement (Fig. 1c) (Red arrow)

According to the data of Table 3; Fig. 2, the consistency of both DE-CMR and PET myocardial perfusion-metabolic imaging to evaluate viable myocardial segments was 90.1% (1379/1530). Based on the Kappa test values the consistency test of two methods was 0.71 (*P* > 0.6), which indicates high consistency of both methods (SE = 0.02, T = 28.14, *p* = 0.000). Among the PET suggested myocardial segments with normal uptake, 14.1% (92/651) of the segments showed different degrees of delayed enhancement, which mainly demonstrated subendocardial enhancement (85.8%, 79/92). The scarred myocardium segments detected by DE-CMR, 15.0% (56/373) had hibernating myocardium.

### Post-CABG heart function improvement

**Fig. 3:**
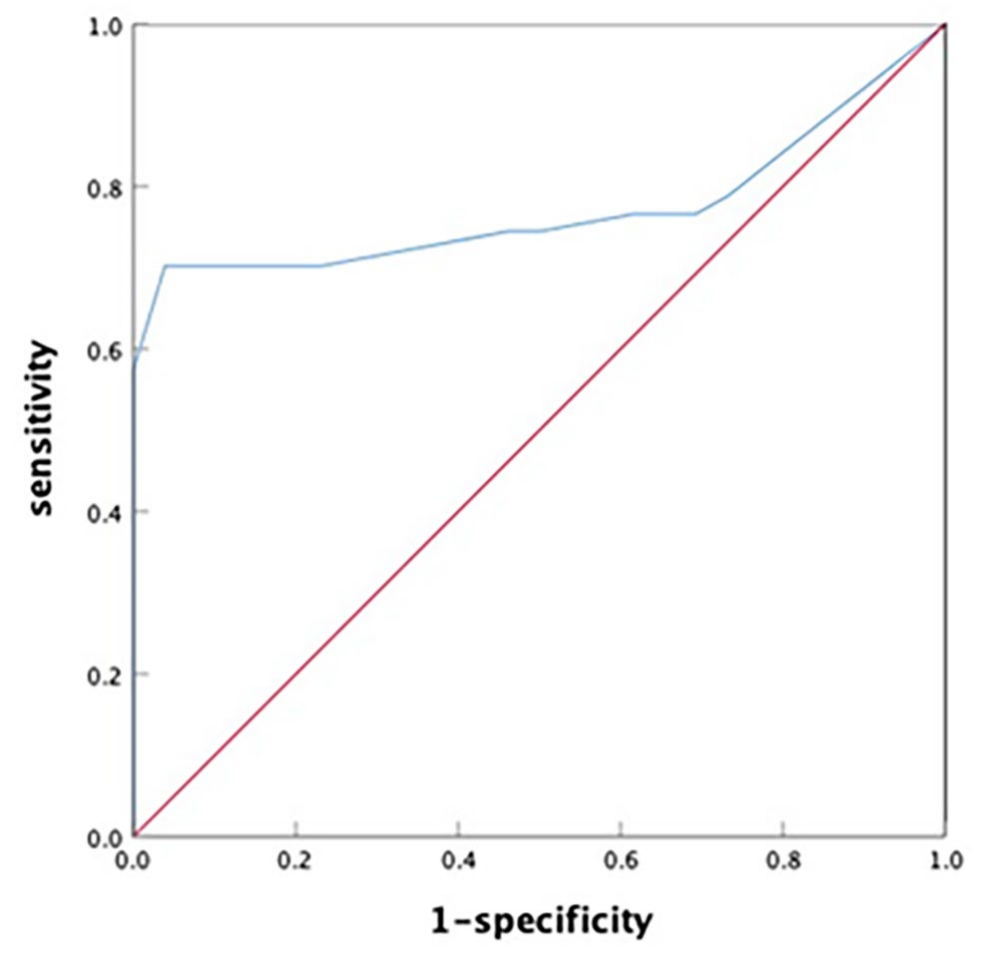
The ROC curve for predicting LVEF improvement after CABG based on number of hibernating myocardium

After CABG, overall heart function improvement was measured in participants. The analyses were performed on data from 73 patients excluding 17 due to left ventricular remodeling at the same time as CABG (Because left ventricular remodeling has a great impact on heart function improvement). The statistics showed in 47 patients (in the △LVEF ≥ 5% group) heart function enhanced after CABG, while 26 patients had no overall heart function improvement (in the △LVEF < 5% group). All parameters in Table 4 were analyzed by univariate analysis, and then only significant parameters were into multivariate analysis. The results showed that the segments of scarred myocardium, the score of delayed enhancement, the percentage of scarred myocardium and hibernating myocardium were independent influencing factors for LVEF improvement after CABG. DE-CMR examination showed that the number of segments with scarred myocardium and overall score of delayed enhancement were significantly lower in the △LVEF ≥ 5% group than the △LVEF < 5% group. Similarly, the PET myocardial imaging results showed that the percentage of scarred myocardium was significantly lower in the △LVEF ≥ 5% group compared to △LVEF < 5% group. In contrast, the percentage of hibernating myocardium was significantly higher in the △LVEF ≥ 5% group than the △LVEF < 5% group. Multivariate logistic regression indicated that number of hibernating myocardium (OR = 1.229, 95%CI: 1.053–1.433, *p* = 0.009) was an independent influencing factor for LVEF improvement in CHD patients post CABG. The AUC of hibernating myocardium was 0.772 (95%CI: 0.662–0.881), and the cut-off value of hibernating myocardium in predicting LVEF improvement was 8.5% (Fig. 3).

**Table 4:** Comparison of the imaging data between the 73 patients in the LVEF improvement group and LVEF non-improvement group

Variable	△LVEF ≥ 5% group (*n* = 47)	△LVEF < 5% group (*n* = 26)	*p* value
Age (years)	62.6 ± 8.1	60.5 ± 11.5	0.435
Sex (M/F)	37/10	19/7	0.601
Diabetes (n, %)	17(36.2)	8(30.8)	0.644
Hypertension (n, %)	22(46.8)	16(61.5)	0.233
Hyperlipidemia (n, %)	13(27.7)	10(38.5)	0.363
Smoking history (n, %)	22(46.8)	12(46.2)	0.958
History of previous PCI (n, %)	30(63.8)	14(53.8)	0.418
AHA heart function grading	2.2 ± 0.7	2.1 ± 0.6	0.472
DE-CMR			
LVEF (%)	31.2 ± 10.4	31.5 ± 9.2	0.923
LVEDV (mL)	234.6 ± 73.6	222.8 ± 68.8	0.497
LVESV (mL)	166.1 ± 68.0	156.3 ± 60.6	0.529
Segment of scarred myocardium (segments)	1 (4)	6(3)	< 0.001
Total score of delayed enhancement (points)	13 (11)	29 (12)	< 0.001
PETmyocardial perfusion-metabolic imaging			
LVEF (%)	32.4 ± 10.9	34.1 ± 8.7	0.467
LVEDV (mL)	180.9 ± 65.1	162.8 ± 57.5	0.226
LVESV (mL)	127.3 ± 59.2	111.1 ± 50.6	0.222
Segment of scarred myocardium (segments)	1 (4)	4(3.5)	< 0.01
Percentage of scarred myocardium (%)	7 (9)	28 (17)	< 0.001
Percentage of hibernating myocardium (%)	16 (23)	5 (6)	< 0.001

## Discussion

This study aimed to investigate the imaging characteristics and consistency of DE-CMR and ^18^F-FDG PET myocardial perfusion-metabolic imaging in evaluating preoperative myocardial viability. The results showed that both DE-CMR and PET myocardial perfusion-metabolic imaging reliably evaluating myocardial viability before CABG. However, PET myocardial perfusion-metabolic imaging has important significance in evaluating the prediction of left heart function improvement. It can determine heart function in severe CHD patients accompanied with heart insufficiency using number of viable myocardium after CABG.

The study compared the degree of delayed enhancement in DE-CMR and the proportion of ^18^F-FDG uptake by myocardium to evaluate myocardial viability in CHD patients. The results indicated high consistency between DE-CMR and PET myocardial perfusion-metabolic imaging (Kappa value = 0.71). Although the consistency of both methods is high but still there were some differences between these two methods. For instance, in this study,.in myocardial segments with CMR delayed enhancement scores of 1 and 2, 14.1% of them were negative results diagnosed by PET myocardial perfusion and metabolism, the efficacy of PET in identifying infarcted myocardium was limited, possibly due to its low spatial resolution, when dealing with small amounts of infarcted myocardium (especially subendocardial infarction). Although PET is widely considered the best method for assessing myocardial viability [21]. However, it may miss diagnosis of subendocardial infarction or even semi-transmural myocardial infarction, which can affect the evaluation of local myocardial movement and overall heart function [22]. In this study, .in myocardial segments with CMR delayed enhancement scores of 3 and 4, 15% of them had hibernating myocardia diagnosed by PET myocardial perfusion and metabolism. Previous studies indicate that about one-third of segments with transmural myocardial infarction have hibernating myocardium [8], that may be attributed to the mixing of necrotic and viable myocardium (hibernating myocardium) during the process of myocardial infarction. .DE-CMR could not accurately identify these hibernating myocardium. In Tables 2 and 45 myocardial segments with CMR delayed enhancement scores of 0 had hibernating myocardia, most of them were located at the junction of normal and scar segments, and were mainly distributed in the subepicardial region. The pathological status of subepicardial myocardium without delayed enhancement cannot be distinguished by CMR, and this part of myocardium can theoretically be any type or mixture of normal myocardium, dormant myocardium or hibernating myocardium. Hibernating myocardium is unstable and fragile, and its presence after myocardial ischemia in CHD is the material basis of myocardial infarction and malignant arrhythmia [23]. Precise identification of hibernating myocardium and timely revascularization could improve the heart function of some patients. In addition, proper treatment of patients with no evident heart function improvement can improve the survival rate to a certain extent [24]. In summary, it is not sufficient to distinguish between viable and infarcted myocardium solely based on the presence and degree of delayed enhancement, and it is necessary to complement this with the advantages of PET in determining hibernating myocardium.

The results of 6–12 months follow-up examination showed the improvement of overall heart function after CABG in 47 patients, while 26 patients had no improvement of overall heart function. The LVEF improvement group had a significantly lower number of scarred myocardial segments, total score of delayed enhancement, and proportion of scarred myocardium compared to the LVEF non-improvement group. However, the proportion of hibernating myocardium was significantly higher in the LVEF improvement group. These results suggested that scarred and hibernating myocardium may be significant factors affecting LVEF improvement after CABG, which is in agreement with the previous studies findings [25]. The outcomes also pointed that the number of hibernating myocardium was the only independent factor influencing the LVEF improvement in CHD patients after CABG.

The analysis of ROC curve showed that the cut-off value of hibernating myocardium proportion was 8.5% in predicting LVEF improvement, indicating that the proportion of hibernating myocardium before CABG is crucial for the postoperative LVEF improvement. It is important to recognize that relying only on the number of hibernating myocardium to predictithe heart function improvement after revascularization is inadequate. Present study identified nine patients with large infarction myocardium but less hibernation myocardium. These patients had highly dilated left ventricle accompanied with a giant ventricular aneurysm. The overall left heart function improvement was noticed after revascularization treatment and ventricular aneurysm folding, the overall function of the left heart also improved in this patient. Other factors that can disturb the left heart function improvement after CABG includes preoperative myocardial ischemia range, degree of left ventricular remodeling, observation time after the procedure, and the success of revascularization [25]. Combination of these two methods to precisely evaluate the myocardium viability, involvement range, and left ventricular structure and function before CABG would provide more detailed data to support the selection procedures and decision making based on the indications.

The study presented some limitations, which are important to consider. Firstly, the study population comprised 90 patients who underwent initial MRI without follow-up MRI. The degree of wall motion abnormality can help to predict functional recovery after PCI [26, 27]. the sample size was relatively small, which may restrict the generalizability of the findings. Secondly, the short follow up time, and especially no moderate- or long- term follow up data was available on MACE events. Moreover, there are ongoing debates regarding the use of myocardial viability in managing patients with ischemic heart failure [28]. To achieve a better understanding and to provide more reliable results it is necessary to conduct research with larger sample sizes, long follow up periods, randomized and control clinical trials.

## Data Availability

All data generated or analysed during this study are included in this published article.
